# A pitfall in hemoglobin A1c measurement with high performance liquid chromatography method in the diagnosis of onset of fulminant type 1 diabetes mellitus

**DOI:** 10.1111/jdi.13834

**Published:** 2022-05-27

**Authors:** Yasuharu Oe, Takatoshi Anno, Yukari Katsuhara, Ryo Sugahara, Miki Kobayashi, Mieko Yakusue, Masayo Tamura, Yukiko Kimura, Fumiko Kawasaki, Kohei Kaku, Hideaki Kaneto, Akira Kitanaka

**Affiliations:** ^1^ Department of Clinical Laboratory Kawasaki Medical School General Medical Center Okayama Japan; ^2^ Department of General Internal Medicine 1 Kawasaki Medical School Okayama Japan; ^3^ Department of Diabetes, Endocrinology and Metabolism Kawasaki Medical School Kurashiki Japan; ^4^ Department of Laboratory Medicine Kawasaki Medical School Kurashiki Japan

## Abstract

We showed a case with the onset of fulminant type 1 diabetes mellitus (FT1DM) whose HbA1c could not be measured with HPLC method. We think that the reason why his HbA1c was not be detected was associated with the presence of labile hemoglobin A1c (LHbA1c). These result mean that it is possible that LHbA1c can bring about a pitfall on HPLC method in diagnosis of onset of FT1DM.

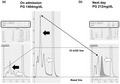

Dear Editor,

Fulminant type 1 diabetes mellitus (FT1DM) is characterized by the acute onset of diabetic ketoacidosis (DKA) soon after the development of typical diabetes symptoms in spite of a near‐normal hemoglobin A1c (HbA1c) level at the onset (HbA1c, <8.7%) and negative serum GAD antibody^1^.

A 59‐year‐old Japanese man was referred to our hospital due to hyperglycemia. He had appetite loss, general fatigue, and repeated vomiting for 3 days. The plasma glucose level was as high as 1,464 mg/dL. HbA1c was not detected with a high performance liquid chromatography (HPLC) method (ADAMS A1c HA‐8182, Arkray Inc., Japan) (Figure 1). Other diabetes‐associated data were as follows: glycoalbumin, 20.6%; total ketone body, 15,610.0 μmol/L; acetoacetate, 3,235.0 μmol/L; β‐hydroxybutyrate, 12,375.0 μmol/L. Blood gas analysis showed severe acidosis: pH, 6.968; base excess, −28.8 mEq/L. Based on such findings, he was finally diagnosed as having diabetic ketoacidosis. Thus, we started whole‐body management, and the hyperglycemia and diabetic ketoacidosis gradually improved.

**Figure 1:**
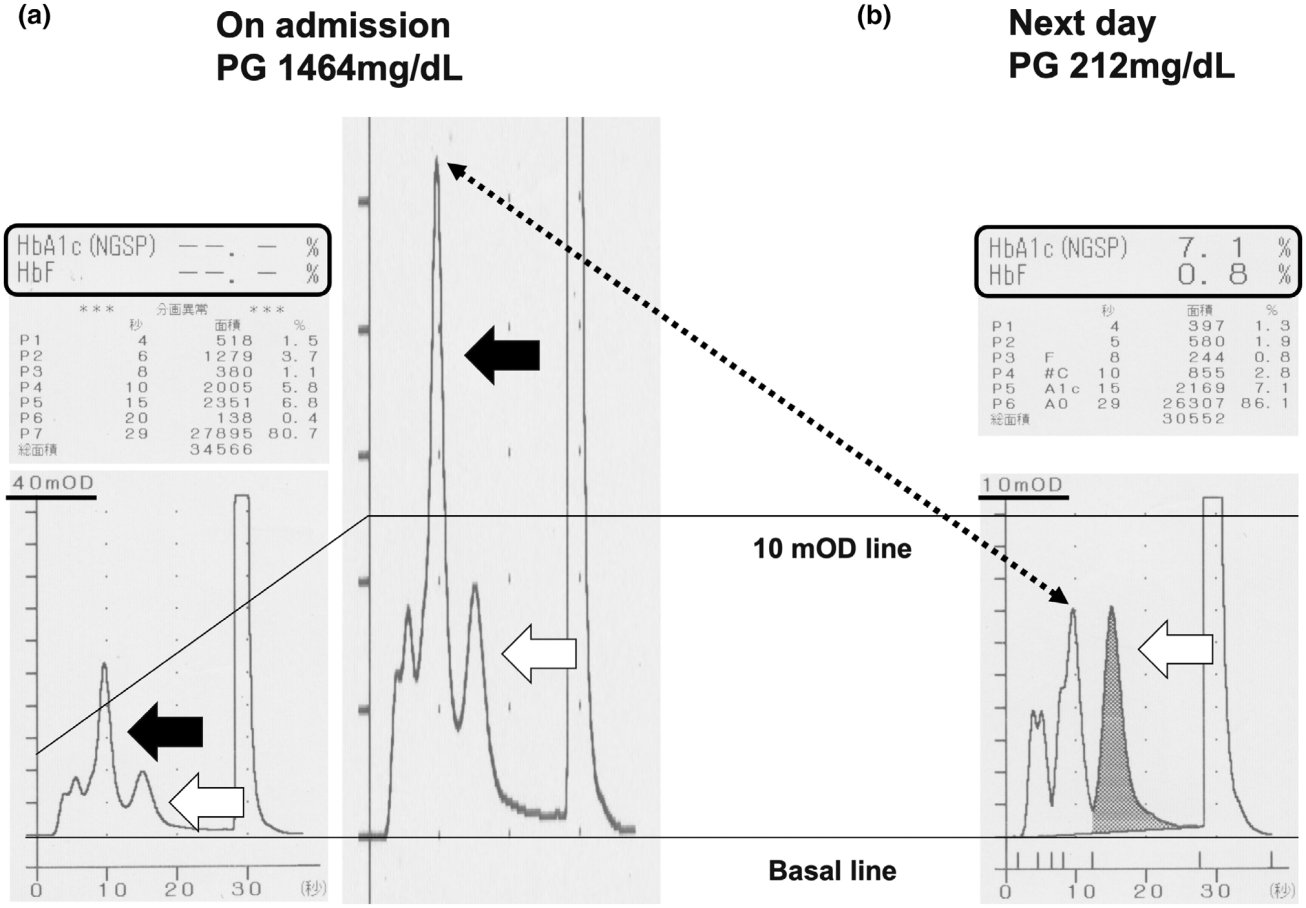
High performance liquid chromatography (HPLC) method in this patient on admission (left and middle panel) and on the next day (right panel). Glycemic hemoglobin A1c (HbA1c) was not detected on admission when the plasma glucose level was as high as 1,464 mg/dL. HbA1c was detected on the next day when the plasma glucose level was decreased to 212 mg/dL. On admission, a very high peak was detected before the HbA1c fraction, which seemed to be labile HbA1c (LHbA1c). On the other hand, the high peak before the HbA1c fraction disappeared on the next day. The scale of the left panel is 40 mOD, and the scale of the right panel is 10 mOD. The middle panel is adjusted with 10 mOD for the left panel. Black arrows show LHbA1c. White arrows show HbA1c. The dotted line shows the fraction including LHbA1c.

Autoantibodies for type 1 diabetes mellitus were all negative. In addition, he suffered from absolute insulin deficiency (immunoreactive insulin, <0.1 μU/mL; C‐peptide immunoreactivity, 0.1 ng/mL; urinary C‐peptide, 9.4, 7.3, 3.6 μg/day). If his HbA1c level had been under 8.7%, we could have diagnosed him as having fulminant type 1 diabetes mellitus. The next day, we re‐examined HbA1c with a HPLC method, and his HbA1c was detected at 7.1% (plasma glucose was 212 mg/dL at that time) (Figure 1b). Moreover, we examined his HbA1c with an enzymatic method using residual serum, with which the HbA1c value was not detected by the HPLC method (plasma glucose was 1,464 mg/dL at that time). His HbA1c was detected at 6.9%. These data clearly show that his HbA1c was not measured with the HPLC method because of severe hyperglycemia. It is noted here that such HbA1c is known as labile hemoglobin A1c (LHbA1c).

The quality of the methods for the determination of HbA1c has been greatly improved over the past decades, and most of the analytical interferences have been controlled^2^. However, HPLC is commonly used for assaying HbA1c, and potential interferences have been described for this method. HbA1c is defined as hemoglobin that is irreversibly glycated at one or both N‐terminal valines of the β‐chain. LHbA1c is a reversible intermediary fraction which is promptly formed between glucose and hemoglobin at an early stage of the glycation reaction^3^. LHbA1c directly depends on the blood glucose concentration and can be increased considerably after the ingestion of carbohydrate. On the other hand, glucose attached to LHbA1c can spontaneously dissociate over time when the ambient glucose level is low. It is also known that LHbA1c is not a single chemical structure but consists of multiple structures with interrelated dissociation kinetics^4^. Afterwards, the bond becomes irreversible, forming stabile HbA1c.

Our patient was in a special condition of having severe hyperglycemia and a near‐normal HbA1c level just after the onset of fulminant type 1 diabetes mellitus. To the best of our knowledge, this is the first report showing that unstable HbA1c interfered with the diagnosis of fulminant type 1 diabetes mellitus. We think that HbA1c was not detected because LHbA1c interfered in this subject with the onset of fulminant type 1 diabetes mellitus. Therefore, while examination of HbA1c is important for the diagnosis of fulminant type 1 diabetes mellitus, it is possible that LHbA1c can interfere in the HPLC method in the diagnosis of the onset of fulminant type 1 diabetes mellitus.

## DISCLOSURE

The authors declare no conflict of interest.

Since this is a case report, but not a clinical study or animal study, ethics approval is not applicable.

Approval of the research protocol: N/A.

Informed consent: Written informed consent was obtained from the patient.

Registry and the registration no. of the study/trial: N/A.

Animal studies: N/A.
